# Evolution and emergence of novel human infections

**DOI:** 10.1098/rspb.2009.1059

**Published:** 2009-08-19

**Authors:** N. Arinaminpathy, A. R. McLean

**Affiliations:** Department of Zoology, Institute for Emerging Infections, James Martin 21st Century School, University of Oxford, Oxford OX1 3PS, UK

**Keywords:** zoonosis, evolution, pandemic

## Abstract

Some zoonotic pathogens cause sporadic infection in humans but rarely propagate further, while others have succeeded in overcoming the species barrier and becoming established in the human population. Adaptation, driven by selection pressure in human hosts, can play a significant role in allowing pathogens to cross this species barrier. Here we use a simple mathematical model to study potential epidemiological markers of adaptation. We ask: under what circumstances could ongoing adaptation be signalled by large clusters of human infection? If a pathogen has caused hundreds of cases but with little transmission, does this indicate that the species barrier cannot be crossed? Finally, how can case reports be monitored to detect an imminent emergence event? We distinguish evolutionary scenarios under which adaptation is likely to be signalled by large clusters of infection and under which emergence is likely to occur without any prior warning. Moreover, we show that a lack of transmission never rules out adaptability, regardless of how many zoonoses have occurred. Indeed, after the first 100 zoonotic cases, continuing sporadic zoonotic infections without onward, human-to-human transmission offer little extra information on pathogen adaptability. Finally, we present a simple method for monitoring outbreaks for signs of emergence and discuss public health implications.

## 1. Introduction

Many novel human infections have zoonotic origins (Morens *et al*. 2004; Wolfe *et al*. 2007; Jones *et al*. 2008). For example, HIV was acquired from African primates (Rambaut *et al*. 2004; Keele *et al*. 2006); SARS coronavirus has been linked to both bats (Li *et al*. 2005) and palm civets (Guan *et al*. 2003); and a recent new arenavirus which killed four out of five cases in Southern Africa is probably derived from rodents (Briese *et al*. 2009). A novel H1N1 influenza A virus, in the early months of a pandemic at the time of writing, was introduced into the human population via swine (Smith *et al*. 2009).

Here we study the epidemiology associated with the establishment of a pathogen in a new host species. As discussed by Antia *et al*. (2003) even a pathogen poorly transmitted among humans, and thus capable only of causing sporadic cases, can acquire adaptations to become capable of sustained human transmission. Such adaptations could arise in response to the selective pressure exerted by the new host environment. Additionally, changing human contact patterns and environmental factors can have the same effect, of enhancing pathogen transmissibility (Woolhouse *et al*. 2005). For example, the avian influenza subtype H5N1 has caused over 400 human cases (World Health Organization 2009), mostly through close contact with infected poultry (Beigel *et al*. 2005). Although it has shown little or no transmission between humans, the possibility of its future adaptation to humans cannot be ruled out.

Previous work on evolving pathogens has studied the effect of host heterogeneity (Yates *et al*. 2006) and pathogen life history (André & Day 2005) on the probability of emergence, per introduction into the human population. Here we ask: what are the epidemiological signs that a pathogen is evolving to adapt for human transmission? For example, under what conditions would such a process be signalled by large outbreaks of infection? Conversely, some pathogens, while capable of infecting humans, may face biological barriers to human adaptation that preclude their ultimate establishment. Is it possible, from case reports, to distinguish such pathogens from those inherently capable of adaptation? Finally, how can case reports be monitored to detect when an ongoing outbreak is about to develop into a full-blown emergence? We approach these questions using simple mathematical models of within-host evolution and between-host transmission. This paper is organized as follows: following a brief discussion of the relationship between pathogen reproductive fitness and outbreak sizes, we present a simple mathematical model of evolution and transmission, and apply it to the questions posed above. We use examples from H5N1 influenza case report data to illustrate the results. Finally, we discuss some public health implications of this work.

## 2. Reproductive fitness and outbreak sizes

**Figure 1.**
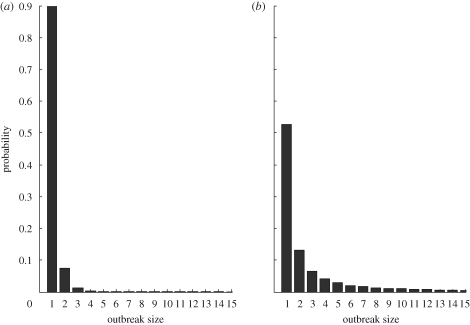
Outbreak size distributions for fixed *R*_0_. (*a*) *R*_0_ = 0.1 and (*b*) *R*_0_ = 0.9. These distributions have mean 1/(1 − *R*_0_).

The basic reproductive number, *R*_0_, is the average number of secondary cases arising from a single infected case, in an otherwise susceptible population (Anderson & May 1991). It is a measure of emergence potential, as *R*_0_ > 1 is a necessary condition for emergence. It is, however, not a sufficient condition, as such pathogens are subject to stochastic extinction with probability 1/*R*_0_^*n*^, where *n* is the number of index cases (May *et al*. 2001). Conversely, infections with *R*_0_ < 1 stutter to extinction with probability 1. Moreover, different regimes for *R*_0_ < 1 show differing epidemiological behaviour in terms of the numbers of cases that outbreaks could involve. Figure 1 shows the probability distributions for outbreak sizes for *R*_0_ = 0.1 (a poorly adapted pathogen) and *R*_0_ = 0.9 (an almost-adapted pathogen). Although neither pathogen is capable of emergence, it is evident that a pathogen with *R*_0_ = 0.9 is capable of causing much larger outbreaks than one with *R*_0_ = 0.1. Indeed, the mean outbreak size for *R*_0_ < 1 is given by 1/(1 − *R*_0_) (Becker 1974).

## 3. The mathematical model

We now incorporate adaptation, using a modified version of a model presented by Antia *et al*. (2003), based on multi-type branching processes. The biological motivation for the model is as follows: an index individual infected with a wild-type (poorly adapted) pathogen is likely to die or recover without infecting anyone else. However, owing to the possibility of the pathogen mutating to acquire some transmission potential, this index case has a non-zero probability of infecting another person. Subsequent adaptations can serve to increase the probability of transmission still further. The stochastic process continues either until extinction of the pathogen, after a certain number of cases, or until emergence of an adapted pathogen. The latter may be subject to yet further adaptations, but we do not consider these stages here. In case of extinction, the process is repeated with another new introduction of a wild-type pathogen from the animal reservoir. In this way, we simulate a series of introductions leading to emergence. Whereas Antia *et al*. (2003) presented a discrete-time model, ours is a continuous-time process as we are also interested in the time course of an outbreak. Moreover, as discussed below, in this model the role of *R*_0_ is to reflect relative probabilities of transmission and recovery; in the context of continuous time, this allows a more natural description of the process of adaptation.

We assume that the host population is sufficiently large to neglect depletion of susceptibles: this is feasible in the early stages of emergence, where there are only comparatively few cases. For simplicity, we also assume that the host population is homogeneous in susceptibility to infection. Furthermore, we assume that the pathogen has to undergo a series of discrete ‘steps’ in order to adapt for human transmission, with each step acquiring an increment in its reproductive number. Thus, we label each adaptive stage with *i*, where *i* = 0 is the wild-type stage and *i* = *n* is the adapted stage. We also have a series of reproductive numbers *R*_0_^(0)^, *R*_0_^(1)^, … , *R*_0_^(^^*n*^^)^, where the wild-type fitness *R*_0_^(0)^ is much less than 1 and only the adapted fitness *R*_0_^(^^*n*^^)^ exceeds 1. Finally, with each adaptive stage we associate a mutation rate, denoted *M*^(^^*i*^^)^. We assume that if infection within a host develops an adaptation, it goes to fixation in that host and is thus the only ‘type’ that can be subsequently transmitted.

Together the parameters *R*_0_^(^^*i*^^)^ and *M*^(^^*i*^^)^ define the stochastic process of evolution and infection as follows. For a given infected individual, there are three possible events that could occur next: transmission, pathogen adaptation, and host recovery/death. These have probabilities *p*_*i*_, *q*_*i*_, *r*_*i*_, respectively, where


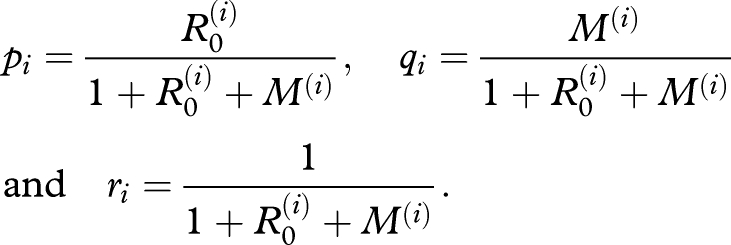


The terms in the denominator arise from rescaling time by the mean infectious period: see appendix in the electronic supplementary material for details of derivations. Using the well-established Gillespie algorithm (Gillespie 1977), this process can be straightforwardly simulated in continuous time. In the case of an infection eventually going extinct, any human cases arising from the index case are collectively a ‘cluster’. We define ‘emergence’ as a situation in which the probability that the outbreak will spontaneously sputter to extinction is less than 10^−6^. Formally, this occurs when there are *m* cases of the adapted strain *R*_0_^(^^*n*^^)^ such that [*R*_0_^(^^*n*^^)^]^*m*^ > 10^6^ (May *et al*. 2001).

## 4. Warning signs of adaptation

**Figure 2.**
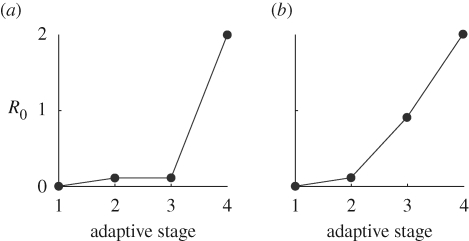
Schematic of (*a*) punctuated and (*b*) gradual scenarios. Parameter values are presented in table 1.

We consider how ongoing pathogen adaptation could be signalled by outbreak sizes, seeking a distribution analogous to figure 1 but with the inclusion of adaptation. Table 1 presents parameters for two different evolutionary scenarios: in the first, ‘punctuated’ scenario, all adaptations are required together for any increase in reproductive fitness. Thus, relative to the wild-type stage, there is little fitness advantage in partial adaptation, and only the adapted stage has a significantly increased fitness. In the second, ‘gradual’ scenario, intermediate adaptations confer incremental improvements in fitness. Figure 2 schematically illustrates the progression in fitness from wild-type to adapted stages for both of these scenarios.

**Table 1.** Parameters for punctuated and gradual scenarios, assuming four adaptive stages. For instance, in the punctuated scenario, the wild-type pathogen has *R*_0_ = 0 in humans. After acquiring an adaptation, it has *R*_0_ = 0.1 and so forth. Here, an adapted pathogen has *R*_0_ = 2, and a mutation rate *M* = 0.1 is associated with all adaptive stages.

scenario	*R*_0_	*M*
punctuated adaptation	[0, 0.1, 0.1, 2]	[0.1, 0.1, 0.1, 0]
gradual adaptation	[0, 0.1, 0.9, 2]	[0.1, 0.1, 0.1, 0]

**Figure 3.**
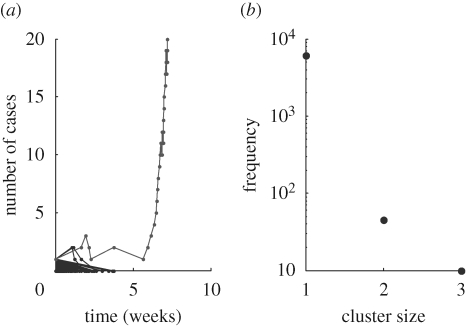
Illustrations of results of the model, punctuated scenario. (*a*) Time series for a series of introductions (black) leading to emergence (grey). All introductions are initiated at time zero. (*b*) Distribution of cluster sizes arising from this calculation.

For illustration figure 3*a* shows sample time courses of infection for a series of introductions leading to an emergence in the punctuated scenario (movies of simulations leading to these outcomes may be found in the electronic supplementary material). Figure 3*b* shows the distribution of cluster sizes arising from this series of introductions. In this example, there were 6173 introductions before emergence. The vast majority of introductions go extinct without transmission (i.e. cluster size 1), but there are some instances where limited transmission has occurred. Only 0.16 per cent of outbreaks exceeded two cases.

**Figure 4.**
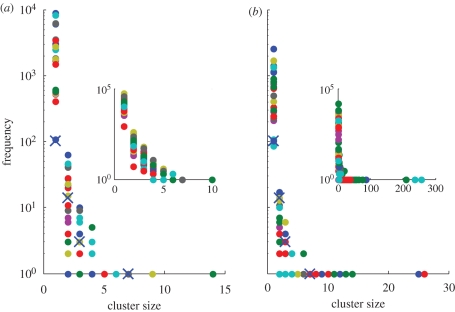
Cluster size distributions for the (*a*) punctuated and (*b*) gradual scenarios. Shown are distributions for 25 independent realizations of the process leading to emergence, with each distribution distinguished by a different colour. Insets show distributions where the penultimate mutation rate has been reduced 10-fold to a value of 0.01. Crosses show H5N1 data from Indonesia.

Figure 4 extends these plots to show the general behaviour of cluster size distributions for both punctuated and gradual scenarios. It displays distributions obtained from 25 independent realisations of the process of emergence, with individual distributions distinguished by colour. Insets show distributions for a 10-fold-reduced mutation rate for the final adaptive step. There is a clear variation between the scenarios in the range of cluster sizes accumulated before emergence: the punctuated scenario causes only small clusters, rarely more than four cases large. The gradual scenario shows a broader range of cluster sizes, and such behaviour is due to the state marginally below pandemic capability (*R*_0_ = 0.9). As indicated by figure 1, such values of *R*_0_ are capable of causing large outbreaks. Indeed, reducing the mutation rate increases the ‘dwell time’ in this state, and thus the greatest range of cluster sizes before emergence is found in the inset of figure 4, gradual scenario. For comparison, crosses in the plots show the distribution of cluster sizes from case reports of H5N1 avian influenza in Indonesia (World Health Organization). As an aside note that, even after more than 400 cases, the pattern of H5N1 clusters appears compatible with both punctuated and gradual scenarios.

For simplicity, we have neglected here the possibility of multiple index cases arising from common exposure to a zoonotic host. Nonetheless, this does not qualitatively alter the results above. The same applies for assuming more steps to emergence (see electronic supplementary material for a discussion of both). Overall, a pathogen with gradually increasing fitness and an adaptive stage marginally below emergence capability is most likely to signal ongoing adaptation by causing large but self-limiting outbreaks. A pathogen undergoing a more punctuated route to emergence is likely to afford less warning.

## 5. Biological barriers to adaptation

If a pathogen causes many hundreds of cases yet fails to show any sustained human transmission, does this mean that it is incapable of adapting for human transmission? More specifically, how many ‘failed’ human cases should occur for such a conclusion?

**Figure 5.**
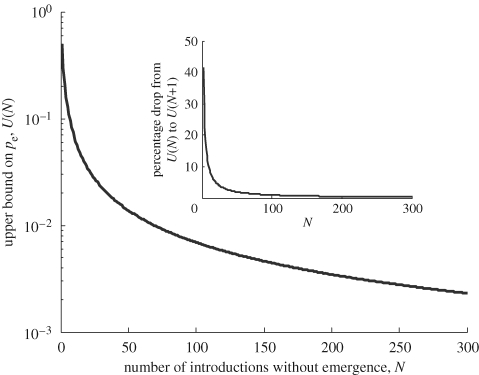
Upper bound on the probability of emergence per introduction *p*_e_, given that *n* introductions have occurred without emergence (*U*(*N*)). Calculated as the maximal value of *p*_e_ giving at least a 50 per cent probability of observing *N* introductions without emergence. Although giving infinitesimally small values for *p*_e_ for large *N*, the curve does not reach *p*_e_ = 0 for any finite *N*. Inset shows the ‘relative information gain’ acquired as *N* increases, measured as the percentage drop from *U*(*N*) to *U*(*N*+1). Again, this quantity has almost entirely diminished by the time *N* = 100.

For a given series of reproductive numbers and mutation rates, it is possible to calculate the probability *p*_e_ of emergence, per introduction, using the theory of multi-type branching processes (Athreya & Ney 1972). If there are insurmountable biological barriers to adaptation, this is equivalent to *p*_e_ = 0. Thus, repeated introductions into the human population are akin to a series of coin tosses, where the probability of ‘heads’ (corresponding to emergence) per toss is *p*_e_. The number of coin tosses before the first head follows a negative binomial distribution. Assume an observation that *N* tosses have yielded only tails, corresponding to a series of failed introductions. The probability of such an outcome is at least 50 per cent as long as
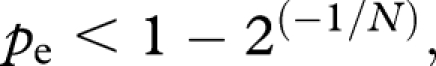
yielding an estimated upper bound on *p*_e_. This upper bound, denoted *U*(*N*), is plotted in figure 5. It shows that, regardless of how many consecutive tails, or failed cases, have occurred, it is never possible to conclude that *p*_e_ = 0. Although mathematically straightforward, in public health terms, this is a simple argument for why a lack of transmission alone gives little information about the adaptability of a pathogen. Indeed, the point is underscored by the behaviour of the punctuated scenario demonstrated above: it is possible for emergence to occur without any prior signs of increasing fitness. Another feature of figure 5 is that *U*(*N*) has diminished by 98 per cent by the time the first 100 cases have occurred. That is, the first 100 cases without emergence indicate a very low probability of emergence per introduction. Beyond this point, continuing failed cases give little more information about the adaptability of the pathogen. The inset of figure 5 plots the percentage drop from *U*(*N*) to *U*(*N*+1) as a function of the number of cases *N*, illustrating that even in relative terms, there is little extra information to be derived from more than 100 cases. In the example of H5N1 influenza, this tally was reached by 8 June 2005 (World Health Organization 2009). Quantitatively, according to this model, sporadic human cases subsequent to that date teach us very little more about whether the virus may ultimately adapt to humans.

## 6. Emergence detection

In practice, it is highly unlikely that the underlying reproductive numbers for an evolving pathogen could be known with any accuracy. Here we focus instead on monitoring ongoing outbreaks for early signs of emergence, the primary concern being rapid notification. We suggest a simple non-parametric method for detection of emergence that does not rely on knowledge of the reproductive fitness parameters. An outbreak in progress is given a ‘rarity score’, with respect to past outcomes, defined as −log_10_(proportion of past outcomes that were more extreme).

**Figure 6.**
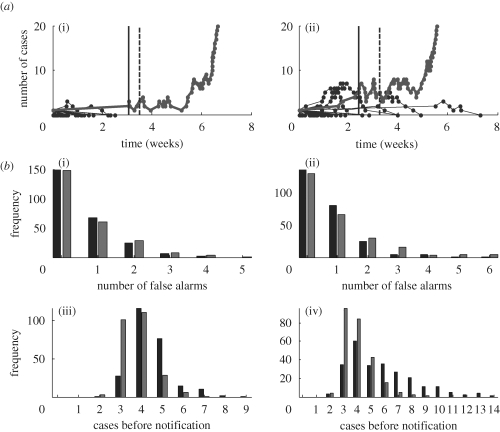
Early warning systems for detecting pandemic emergence. The ‘single’ method monitors outbreak size, raising an alarm if a threshold with respect to past outbreak sizes is exceeded. The ‘double’ method additionally monitors daily incidence (see text for details). (*a*) Sample time courses of infection leading to emergence. Vertical, dashed line: time of notification with the single approach. Vertical, solid line: time of notification with the double approach. (i) punctuated scenario (ii) gradual scenario. (*b*) Graphs of algorithm performance, calculated over 250 simulated emergences, with the alarm silenced for the first 400 introductions. Black bars, single; grey bars, double. (i, ii) Specificity is measured by number of false alarms before an emergence, (iii, iv) while sensitivity is measured by the number of cases before an alarm occurs, in the event of a genuine emergence. Left- and right-hand panels refer to punctuated and gradual scenarios, respectively.

We compare two approaches: for the ‘single rarity’ approach, a record is kept of the total outbreak size from every introduction. For an outbreak in progress, a rarity score can then be calculated for the cumulative number of cases. When this exceeds a given threshold, the alarm is triggered. The ‘double rarity’ approach likewise monitors outbreak sizes, and additionally monitors the daily incidence. A record is kept of the greatest incidence reached following every introduction, and thus a rarity score is calculated, in real time, for an outbreak in progress. An alert is then triggered where at least one rarity score exceeds a given threshold. Specificity is measured by the number of introductions causing a false alarm (introductions causing an alarm and subsequently going extinct). Sensitivity is measured, in the event of a true emergence, by the number of cases before the first alarm. Figure 6 shows measures of algorithm performance, calculated over 250 emergences simulated as described above, under both the gradual and punctuated scenarios. Both single and double approaches show a similar performance under the punctuated scenario. However, under the gradual scenario, the double approach shows an appreciably better performance in sensitivity, also doing so more consistently. It raises the alarm after a mean (standard deviation) of 4.2 (1.3) cases, compared with 5.8 (2.4) for the single approach. As might be expected, this comes at the expense of some specificity: the single approach causes a mean (standard deviation) of 0.68 (0.97) false alarms before emergence, compared with 1.0 (1.3) for the double method. In practice, epidemiological investigation and contact tracing will be essential for identifying which cases are epidemiologically linked. Furthermore, we do not address here the timing or distribution of index cases. Nonetheless, while more sophisticated schemes are undoubtedly possible, these results illustrate how additional outbreak characteristics, such as incidence, could contribute to emergence detection.

## 7. Discussion and conclusion

The work presented here underlines the unpredictability of infectious diseases undergoing adaptations for human transmission. Nonetheless, it also illustrates that general patterns of epidemiological behaviour can be associated with different evolutionary pathways. The scenarios presented here have widely different implications for public health, and each presents its unique problem in terms of containment. A pathogen following a punctuated route to adaptation is liable to emergence without any warning. Moreover, under this scenario, large outbreaks tend to be caused only by adapted pathogens, and so such outbreaks will be comparatively difficult to contain. By contrast, the gradual route offers some warning of adaptation in the form of large but self-limiting outbreaks. Both these clusters and the early stages of emergence are composed mainly of cases of a partially adapted pathogen, rendering containment comparatively easier. However, a danger is that repeated false alarms would elicit repeated containment efforts, potentially draining valuable resources.

There are many possible refinements to the simple model of transmission presented here. One shortfall in the model is where large numbers of infection are predicted: in reality, large outbreaks will tend to be limited by local depletion of susceptibles, as well as being likely to trigger spontaneous social distancing and public health interventions. Host heterogeneity may also play a role. For example, Lloyd-Smith *et al*. (2005) point out in the context of a non-adapting pathogen that individual variation in transmissibility, hidden by a population-level value for *R*_0_, can have a strong effect on the outcomes of introductions, making extinction more likely than in a homogeneous population. Yates *et al*. (2006) explore different types of heterogeneity, including susceptibility to infection, and make the elegant distinction that, for non-adapted stages, the rate of adaptation has a stronger effect on probability of extinction than heterogeneity, and conversely for adapted stages. Extending this discussion to cluster sizes before emergence, these and other aspects of host population structure would be areas for refinement in models more detailed than those we have presented here. Nonetheless, the general insights offered by our approach are likely to remain valid. While mathematical models can be no replacement for detailed epidemiological investigations in the field, such as contact tracing and laboratory analysis, we hope we have shown here that they can offer valuable, objective insights into potential pre-emergence scenarios. Together with established frameworks for rapid case identification and management, mathematical models can play an important role in our toolkit for preparedness in public health.
